# Acute effects of nicotine-free and flavour-free electronic cigarette use on lung functions in healthy and asthmatic individuals

**DOI:** 10.1186/s12931-017-0518-9

**Published:** 2017-02-10

**Authors:** Marie-Ève Boulay, Cyndi Henry, Ynuk Bossé, Louis-Philippe Boulet, Mathieu C. Morissette

**Affiliations:** 1 Quebec Heart and Lung Institute - Université Laval, 2725 Chemin Sainte-Foy, Quebec City, PQ G1V 4G5 Mallet M2679 Canada; 2 Department of Medicine, Université Laval, Quebec City, Canada

## Abstract

We designed a crossover and placebo-controlled trial to investigate the impact of a 1-h acute vaping session of nicotine-free and flavour-free e-liquid on the pulmonary functions and respiratory mechanics of healthy and asthmatic individuals. This study shows that a 1-h vaping session of a high-grade and contaminant-free mixture of propylene glycol and glycerol using a commercially available electronic cigarette performed in a controlled environment does not significantly impact pulmonary functions, respiratory mechanics or symptoms in healthy or asthmatic subjects.

To the Editor,

Electronic cigarette (e-cigarette) use or ‘vaping’ has stormed the world over the past years. It is available everywhere and regardless of age, and is aggressively marketed as a safe way to stop smoking despite a lack of solid evidence. Reality is that we do not know the consequences of vaping on pulmonary health and research is urgently needed to identify potential health hazards.

While most emphasis is placed on nicotine and flavouring agents found in e-liquid, very little scientific knowledge is available on the acute effects of propylene glycol and glycerol inhalation, the two compounds representing the great majority of e-liquid total volume. We therefore aimed at characterizing the acute effects of inhaling propylene glycol and glycerol on lung functions. We were also interested to compare potential differences between volunteers with no documented lung disease and volunteers with diagnosed asthma, as the latter are generally more sensitive to pulmonary irritants and bronchoconstrictive stimuli.

We conducted a randomized cross-over, placebo-controlled study on the acute effect of nicotine-free and flavour-free e-cigarette vapor inhalation. Volunteers were aware of using full (experimental) or empty (placebo) e-cigarettes as no vapors were coming out of the empty placebo device. The e-liquid consisted of a mixture of 70% USP-grade propylene glycol (PG) and 30% USP-grade glycerol (Gly), mixed in our laboratory under a biosafety cabinet. This 70%PG/30%Gly mixture is largely representative of what is used on the market to dissolve nicotine and/or flavours. Our approach was to create the best-case scenario, where unwanted and possibly harmful contaminants are absent, to specifically investigate the pulmonary effects of PG and Gly. Twenty healthy volunteers and ten asthmatic volunteers were recruited for the trial. Healthy volunteers were aged between 20 and 37 years, had no documented lung disease and had normal response to methacholine. Asthmatic volunteers were aged between 21 and 40 years and all had received a diagnosis of asthma and had airway hyperresponsiveness as shown by a positive methacholine challenge. All volunteers were non-smokers and none were active e-cigarette users. Moreover, none of the volunteers were exposed to secondary tobacco or e-cigarette vapors at home. All volunteers gave their written informed consent and the IUCPQ-UL Ethics Board approved the trial (reference number 2015–2459 21256).

The experimental and placebo sessions took place 1 week apart. A preceding visit was also planned, where baseline measurements were taken (i.e. vital signs, lung functions, airway resistance measurements), a methacholine challenge was performed according to Juniper et al. [1], and volunteers were familiarized with the e-cigarette. On the following two sessions (experimental and placebo), volunteers were asked to inhale three times per minute, in sitting position, for a total duration of 1 hour. Respiratory mechanics and lung functions were measured by the forced oscillation technique (TremoFlow) and spirometry, respectively and in that order, immediately before (T0), immediately after (T60), and 30 min after (T90) the inhalation sessions. Respiratory symptoms were collected according to the Borg perception scale at the same time points as well as every 20 min during the inhalation session. The fraction of exhaled nitric oxide (FeNO) and serum C-reactive protein (CRP) were also measured in asthmatic volunteers. FeNO and serum samples were not collected in non-asthmatic volunteers.

All volunteers were able to complete the sessions. Data collected are presented in Table 1. A few subjects noticed symptoms when using the loaded e-cigarette including cough, chest tightness, and secretions, but no consensual differences in symptoms were noticed in healthy or asthmatic volunteers. Changes in vital signs such as oxygen saturation, heart rate, and breathing frequency were not different between placebo or experimental sessions. No changes in lung functions were observed using either spirometry or forced oscillation technique. Finally, signs of flaring inflammation, such as increased FeNO and serum CRP levels, were not observed in response to e-cigarette in the asthmatic group.

**Table 1:** Symptoms, vital signs, lung function and inflammation parameters at baseline and following a 60-min period of air or e-liquid vaping in non-asthmatic and asthmatic subjects

	Healthy Volunteers (*N* = 20)						Asthmatic Volunteers (*N* = 10)					
	Placebo (no e-liquid)			Experimental (with e-liquid)			Placebo (no e-liquid)			Experimental (with e-liquid)		
	T0^a^	T60^a^	Mean difference (95% CI Lower-Upper)	T0^a^	T60^a^	Mean difference (95% CI Lower-Upper)	T0^a^	T60^a^	Mean difference (95% CI Lower-Upper)	T0^a^	T60^a^	Mean difference (95% CI Lower-Upper)
Symptoms												
Cough	0.1 ± 0.2	0.1 ± 0.2	0.025 (0.021 to 0.029)	0.1 ± 0.2	0.2 ± 0.5	0.10 (0.09 to 0.11)	0.1 ± 0.1	0 ± 0	−0.05 (−0.05 to − 0.05)	0.3 ± 0.9	0 ± 0	−0.30 (−0.32 to − 0.28)
Chest tightness	0 ± 0	0.1 ± 0.2	0.050 (0.047 to 0.053)	0 ± 0	0.4 ± 1.1	0.38 (0.36 to 0.39)	0.1 ± 0.2	0.1 ± 0.2	0 (−0.01 to 0.01)	0 ± 0	0.6 ± 0.8	0.60 (0.58 to 0.62)
Breathlessness	0 ± 0.1	0 ± 0.1	0 (−0.002 to 0.002)	0.1 ± 0.2	0.2 ± 0.7	0.10 (0.09 to 0.11)	0.1 ± 0.2	0 ± 0	−0.10 (−0.10 to − 0.10)	0.2 ± 0.4	0.2 ± 0.2	−0.05 (−0.06 to − 0.04)
Secretions	0.1 ± 0.2	0.1 ± 0.2	0 (−0.004 to 0.004)	0.1 ± 0.2	0.3 ± 0.7	0.25 (0.24 to 0.26)	0 ± 0	0.2 ± 0.3	0.20 (0.19 to 0.21)	0.4 ± 0.7	0.3 ± 0.6	−0.10 (−0.11 to − 0.09)
Wheezing	0 ± 0	0 ± 0	0 (0 to 0)	0 ± 0	0.1 ± 0.2	0.05 (0.05 to 0.05)	0 ± 0	0 ± 0	0 (0 to 0)	0.1 ± 0.3	0 ± 0	−0.10 (−0.11 to − 0.09)
Heart rate (beat/min)	69 ± 9	63 ± 8	−6.4 (−6.5 to − 6.2)	69 ± 9	61 ± 10	−11.4 (−11.6 to − 11.1)	74 ± 10	69 ± 11	−5.2 (−5.3 to − 5.1)	78 ± 9	74 ± 10	−4.3 (−4.4 to − 4.2)
Saturation (% O_2_)	98 ± 2	96 ± 7	0.25 (0.23 to 0.27)	97 ± 3	98 ± 1	0.80 (0.77 to 0.83)	98 ± 1	99 ± 1	0.40 (0.38 to 0.42)	98 ± 1	98 ± 1	0.70 (0.68 to 0.72)
Respiratory rate (respiration/min)	16 ± 3	15 ± 3	−0.80 (−0.83 to − 0.77)	15 ± 3	15 ± 3	0.20 (0.15 to 0.25)	14 ± 3	14 ± 3	0 (−0.04 to 0.04)	17 ± 3	15 ± 3	−1.80 (−1.84 to − 1.76)
Spirometry												
FEV_1_ (L)	4.0 ± 0.7	4.0 ± 0.7	0.021 (0.020 to 0.021)	3.9 ± 0.7	3.9 ± 0.7	−0.021 (−0.022 to − 0.020)	3.4 ± 0.4	3.4 ± 0.4	−0.01 (−0.01 to − 0.01)	3.4 ± 0.4	3.4 ± 0.4	0.014 (0.013 to 0.015)
FVC (L)	4.9 ± 0.9	4.9 ± 0.9	0.004 (0.002 to 0.005)	4.9 ± 0.9	4.9 ± 0.9	−0.043 (−0.044 to − 0.041)	4.4 ± 0.8	4.3 ± 0.8	−0.05 (−0.05 to − 0.05)	4.4 ± 0.8	4.3 ± 0.8	−0.069 (−0.070 to − 0.068)
FEV_1_/FVC	0.80 ± 0.05	0.81 ± 0.05	0.010 (−0.016 to 0.036)	0.80 ± 0.05	0.81 ± 0.05	0.314 (0.296 to 0.332)	0.78 ± 0.09	0.79 ± 0.08	−1.10 (−1.13 to − 1.11)	0.78 ± 0.08	0.79 ± 0.08	1.550 (1.516 to 1.584)
FOT												
R_5_ (cm H_2_O.s/L)	2.656 ± 0.595	2.778 ± 0.688	0.121 (0.116 to 0.126)	2.766 ± 0.702	2.564 ± 0.633	−0.201 (−0.212 to − 0.190)	3.858 ± 0.717	3.812 ± 0.768	−0.046 (−0.054 to − 0.038)	3.583 ± 0.593	3.804 ± 0.591	0.221 (0.214 to 0.227)
R_19_ (cm H_2_O.s/L)	2.717 ± 0.538	2.766 ± 0.597	0.048 to (0.044 to 0.053)	2.792 ± 0.592	2.739 ± 0.471	−0.054 (−0.057 to − 0.050)	3.715 ± 0.478	3.664 ± 0.531	−0.051 (−0.055 to − 0.046)	3.639 ± 0.645	3.711 ± 0.496	0.071 (0.067 to 0.076)
R_5–19_ (cm H_2_O.s/L)	−0.061 ± 0.211	0.012 ± 0.225	0.073 (0.071 to 0.075)	−0.027 ± 0.309	−0.175 ± 0.468	−0.147 (−0.157 to − 0.138)	0.143 ± 0.364	0.148 ± 0.368	0.005 (0.001 to 0.009)	−0.056 ± 0.208	0.093 ± 0.255	0.149 (0.144 to 0.154)
X_5_ (cm H_2_O.s/L)	−0.841 ± 0.282	−0.866 ± 0.290	−0.025 (−0.027 to − 0.023)	−0.911 ± 0.456	−0.862 ± 0.386	0.050 (0.048 to 0.052)	−1.329 ± 0.461	−1.230 ± 0.428	0.029 (0.026 to 0.032)	−1.118 ± 0.286	−1.029 ± 0.250	0.089 (0.086 to 0.091)
Fres (Hertz)	10.765 ± 1.844	11.157 ± 2.161	0.392 (0.373 to 0.410)	11.597 ± 3.844	11.140 ± 3.319	−0.457 (−0.468 to − 0.446)	14.269 ± 4.026	13.756 ± 3.596	−0.514 (−0.555 to − 0.472)	13.375 ± 3.319	12.860 ± 2.422	−0.515 (−0.547 to − 0.483)
Ax (cm H_2_O/L/s^a^Hz)	2.597 ± 1.618	2.812 ± 1.694	0.216 (0.203 to 0.228)	3.538 ± 5.028	3.150 ± 4.134	−0.389 (−0.403 to − 0.375)	5.798 ± 3.513	5.510 ± 3.078	−0.289 (−0.328 to − 0.249)	4.557 ± 2.411	4.040 ± 2.018	−0.517 (−0.534 to − 0.499)
IC (L)	2.698 ± 0.694	2.632 ± 0.731	−0.046 (−0.050 to − 0.042)	2.728 ± 0.628	2.697 ± 0.635	−0.019 (−0.021 to − 0.016)	2.616 ± 0.660	2.569 ± 0.695	−0.047 (−0.053 to − 0.040)	2.894 ± 0.708	2.701 ± 0.785	0.096 (0.086 to 0.107)
TV (L)	0.837 ± 0.346	0.904 ± 0.364	0.067 (0.066 to 0.069)	0.890 ± 0.372	0.925 ± 0.422	0.035 (0.033 to 0.036)	0.975 ± 0.297	0.942 ± 0.392	−0.033 (−0.041 to − 0.025)	0.983 ± 0.308	1.090 ± 0.422	0.106 (0.101 to 0.111)
FeNO (ppb)	ND	ND	ND	ND	ND	ND	25 ± 11	24 ± 11	0.40 (0.34 to 0.46)	23 ± 12	21 ± 12	0.20 (0.13 to 0.27)
CRP (mg/L)	ND	ND	ND	ND	ND	ND	1.31 ± 1.38	1.00 ± 0.89^b^	−0.47 (−0.50 to − 0.44)	1.89 ± 2.18	1.93 ± 2.22^b^	0.039 (0.038 to 0.040)

Data are presented as mean ± standard deviation (SD)

^a^ T0 represents data recorded right before and T60 represents data recorded right after a 60-min period of vaping, ^b^ Data recorded 30 min after a 60-min period of vaping (T90)

Abbreviations: *Ax* reactance area, *BMI* body mass index, *CRP* C-reactive protein, *FeNO* exhaled nitric oxide, *FEV*_1_ forced expiratory volume in 1 second, *FOT* forced oscillation technique, *FVC* forced vital capacity, *Fres* resonant frequency, *IC* inspiratory capacity, *R*_5_ airway resistance at 5Hz, *R*_19_, airway resistance at 19 Hz, *SD* standard deviation, *TV* tidal volume, *X*_5_ airway reactance at 5 Hz

This study shows that a 1-h inhalation session of a high-grade and contaminant-free mixture of propylene glycol and glycerol using a commercially available e-cigarette performed in a controlled environment does not significantly impact pulmonary function or symptoms in both healthy and asthmatic subjects. This is consistent with *Ferrari et al.* [2] and *Flouris et al.* [3]. The latter showed in healthy individuals that inhalation sessions of 5 or 30 min, respectively, had no significant effects on lung functions measured by spirometry.

An unavoidable limitation of this study and similar studies is that individuals are aware of using the full or empty e-cigarette. It is simply impossible to blind the individuals since the e-liquid has an off-taste and produces a significant amount of visible vapors. Subjective measures such as symptoms could have been affected by this limitation, but we believe that the objective physiological measures (i.e. spirometry, forced oscillation technique) were not affected.

Therefore, while acute exposure to e-cigarette vapors does not seem to cause significant functional pulmonary alterations in healthy and asthmatic individuals, there is an urgent need to study the effects of chronic use, especially in more susceptible populations such as teenagers, smokers, and individuals with chronic lung diseases such as asthma and chronic obstructive pulmonary disease.

## Abbreviations

Ax: Reactance area
BMI: Body mass index
CRP: C-reactive protein
E-cigarette: Electronic cigarette
FeNO: Fraction of exhaled nitric oxide
FEV_1_: Forced expiratory volume in 1 second
FOT: Forced oscillation technique
Fres: Resonant frequency
FVC: Forced vital capacity
IC: Inspiratory capacity
R_19_: Airway resistance at 19 Hz
R_5_: Airway resistance at 5Hz
SD: Standard deviation
TV: Tidal volume
X_5_: Airway reactance at 5 Hz
